# Virtual Reality in the Preoperative Planning of Adult Aortic Surgery: A Feasibility Study

**DOI:** 10.3390/jcdd9020031

**Published:** 2022-01-18

**Authors:** Djamila Abjigitova, Amir H. Sadeghi, Jette J. Peek, Jos A. Bekkers, Ad J. J. C. Bogers, Edris A. F. Mahtab

**Affiliations:** Department of Cardiothoracic Surgery, Erasmus University Medical Center, Room Rg-619, P.O. Box 2040, 3000 CA Rotterdam, The Netherlands; h.sadeghi@erasmusmc.nl (A.H.S.); j.j.peek@erasmusmc.nl (J.J.P.); j.a.bekkers@erasmusmc.nl (J.A.B.); a.j.j.c.bogers@erasmusmc.nl (A.J.J.C.B.); e.mahtab@erasmusmc.nl (E.A.F.M.)

**Keywords:** virtual reality, ascending aorta, aortic arch surgery, surgical planning

## Abstract

*Background:* Complex aortic anatomy needs careful preoperative planning in which a patient-tailored approach with novel immersive techniques could serve as a valuable addition to current preoperative imaging. This pilot study aimed to investigate the technical feasibility of virtual reality (VR) as an additional imaging tool for preoperative planning in ascending aortic surgery. *Methods:* Ten cardiothoracic surgeons were presented with six patients who had each undergone a recent repair of the ascending aorta. Two-dimensional computed tomography images of each patient were assessed prior to the VR session. After three-dimensional (3D) VR rendering and 3D segmentation of the ascending aorta and aortic arch, the reconstructions were analyzed by each surgeon in VR via a head-mounted display. Each cardiothoracic surgeon completed a questionnaire after each planning procedure. The results of their assessments were compared to the performed operations. The primary endpoint of the present study was a change of surgical approach from open to clamped distal anastomosis, and vice versa. *Results*: Compared with conventional imaging, 80% of surgeons found that VR prepared them better for surgery. In 33% of cases (two out of six), the preoperative decision was adjusted due to the 3D VR-based evaluation of the anatomy. Surgeons rated CardioVR usefulness, user-friendliness, and satisfaction with median scores of 3.8 (IQR: 3.5–4.1), 4.2 (IQR: 3.8–4.6,) and 4.1 (IQR: 3.8–4.7) on a five-point Likert scale, respectively. *Conclusions:* Three-dimensional VR imaging was associated with improved anatomical understanding among surgeons and could be helpful in the future preoperative planning of ascending aortic surgery.

## 1. Introduction

Aortic surgery remains a complex and challenging field. Each outcome is closely related to surgical skill and, consequently, to the available caseload [1]. Adequate preoperative planning is an essential surgical skill that can decrease unnecessary additional interventions such as deep hypothermic circulatory arrest (DHCA), which can contribute to a higher rate of complications [2,3]. Computed tomography (CT), magnetic resonance imaging (MRI), and echocardiography, together with three-dimensional (3D) anatomical reconstructions, are common imaging techniques to help surgeons previsualize a surgical intervention to define the surgical approach in the context of aortic surgery. However, these existing imaging tools are sometimes insufficient to provide a complete and detailed view, causing alternations between preoperative and operative decisions. Virtual reality is (VR) an emerging and promising technique that enables the creation of digital objects and virtual animations in an immersive digital environment that can be visualized and interacted with via head-mounted displays (HMD’s) and controllers. In the field of cardiovascular surgery, an increasing number of reports have become available to demonstrate the potential benefits of VR for education, surgical planning, and simulation [4]. The preoperative planning of ascending aortic repair can be challenging due to the extent of the disease with regard to the arch. A close proximity to the aortic arch makes the decision to operate on a clamped aorta less likely, consequently significantly changing the invasiveness of the surgery and operative strategy. Previous studies have shown that surgical errors can be reduced through the effective use of immersive VR to visualize patient anatomy [5,6]. The ability to properly visualize the complex spatial 3D anatomy of the ascending aorta and arch can potentially change the surgical approach, reduce operating room time, and ensure better surgical outcomes. Therefore, we aimed to assess the technical feasibility, usefulness, and effectiveness of VR for the operative planning of ascending aortic surgery and the routine necessity of an open distal anastomosis during these procedures.

## 2. Materials and Methods

### 2.1. Patient Selection

Six patients who had each recently undergone a surgical replacement of the ascending aorta were selected for this study. The Medical Ethics Committee of Erasmus University Medical Center in Rotterdam granted approval for this study (MEC-2020-0107).

### 2.2. Computed Tomography

To create volumetric 3D VR renderings of the CT scans, preoperative scans were essential. Except for a maximum of 1000 CT images, there were no specific technical requirements for the CT scans; however, an appropriate quality for conventional two-dimensional (2D) review was necessary. All patients underwent a contrast-enhanced CT scan of the thorax and abdomen. Scans were performed on a variety of multislice CT scanner models with a minimal 64-multidetector configuration, consisting of both ECG-triggered and non-ECG-triggered scan modes. Scans were performed with iodinated intravenous contrast media and optimized for CT-angiography with thin collimations. Doses were optimized to patient body habitus by automated tube current modulation on all scanners, with additional tube voltage modulation to a vascular exam type on some. From thin raw data, a variety of image reconstructions were performed in axial, coronal, and sagittal views. Initially, all the surgeons evaluated the scans of all the patients by conventionally viewing the CT scans in different views and noted their operative plans. Afterwards, 3D VR reconstructions of the same patients were reviewed by the surgeons and, again, the surgeons noted their operative plans. Double-oblique measurements were performed in a 2D transverse plane perpendicular to the centerline in the sagittal plane in order to compute the true cross-sectional diameter of each aorta.

### 2.3. 3D Image Segmentation

To generate 3D segmentations of each ascending aorta and aortic arch, digital imaging and communications in medicine (DICOM) files of CT scans were extracted from the patient archiving and communication system (PACS) and, after anonymization, loaded into ITK-SNAP14 software [7]. To create a VR model reconstruction, segmentation of each patient’s ascending aorta, arch vessels, and proximal descending aorta was performed semi-automatically, utilizing gray value thresholding, region-growing algorithms, and manual editing using 3D Slicer software [8]. Three-dimensional segmentations of the aforementioned structures were exported as NIfTI (Neuroimaging informatics Technology Initiative (nii.gz.)) files that were directly loaded, in combination with the DICOM CT images, as 3D VR reconstructions using a CardioVR workstation (MedicalVR, Amsterdam, The Netherlands).

### 2.4. Immersive 3D VR Rendering and Preoperative Planning

Anonymized DICOM files, together with 3D segmentation files, were loaded into our CardioVR surgical planning tool. CardioVR was developed in collaboration with MedicalVR B.V. (Amsterdam, The Netherlands). CardioVR was constructed utilizing Unity Software. It enables immediate automatic CT to 3D VR rendering and provides the user with additional editing tools with which to view the conventional 2D CT scan images, change visual scan settings (e.g., opacity), highlight structures by brushing (coloring) and erasing parts of the scan, and add/remove/highlight the color of additional 3D segmentations. All scans could be accurately reviewed with an HMD (Oculus Rift S, Oculus VR, Irvine, CA, USA) and controllers, while 3D projections of the VR view were provided on a computer screen.

### 2.5. Data Acquisition and Questionnaire Design

**Figure 1:**
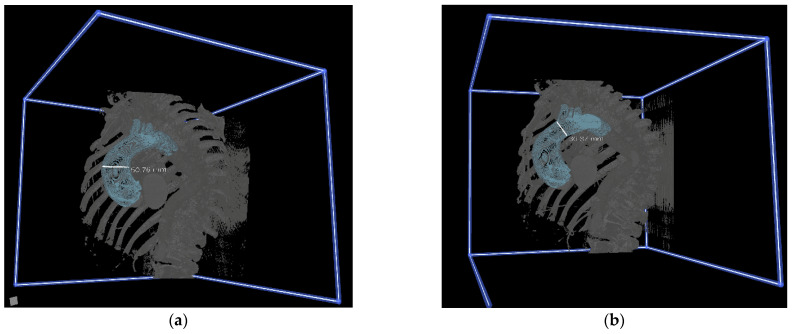

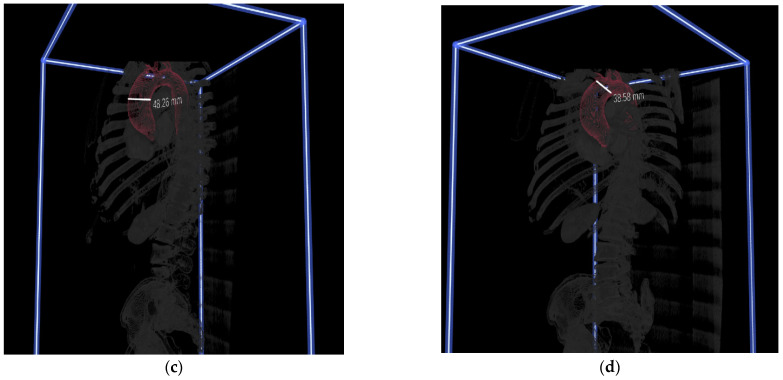
Screen captures of 3-dimensional (3D) virtual reality (VR) rendered images. Evaluations and measurements of 3D reconstructions of ascending aortas and arches in 3D VR for preoperative planning. (**a**,**c**) Measurements of maximal ascending aortic diameter. (**b**,**d**) Measurements of aortic diameter proximal to the brachiocephalic artery.

First, ten cardiothoracic surgeons who had engaged in performing aortic surgery were asked to complete a standardized self-reported questionnaire to evaluate their expectations of VR simulation. Afterward, they individually evaluated the preoperative CT images of six patients, first in 2D CT and then in immersive 3D VR. They were asked to measure the maximal diameter of the ascending aorta and the diameter of the ascending aorta just before the brachiocephalic artery (Figure 1, Video S1). Next, all surgeons were presented with a second questionnaire to assess their preoperative preparation and decision making, the perceived quality of the conventional imaging, and the perceived knowledge gain with the VR. Another questionnaire, based on validated instruments, to measure the usefulness, ease of use, and effectiveness of, as well as their satisfaction with, CardioVR was filled out by all surgeons at the end [9]. All these questionnaires were composed based on a series of standards to measure the validity of the developed CardioVR surgical planning tool [10]. Response options ranged from “strongly disagree” (1 point) to “strongly agree” (5 points) on a five-point Likert scale. Complete questionnaires are provided in the Supplementary Material.

### 2.6. Statistical Analysis

Analyses of clinical data were performed in Microsoft Office Excel 2016 (Microsoft, Redmond, WA, USA) and in IBM SPSS Statistics for Windows, version 25 (IBM Cor., Armonk, NY, USA). Continuous data are presented as mean + SD, or median and IQR, and categorical data (including Likert scales) are presented as proportions and/or counts. Paired comparisons of Likert scale responses were made using the Wilcoxon signed-rank test. All tests were two tailed and statistical significance was inferred at *p* < 0.05.

## 3. Results

### 3.1. Patient Characteristics and Surgeons’ Attitudes towards VR before the Study

The six patients selected for this study underwent repair of their ascending aorta. Five of these patients underwent an aortic root replacement either with a mechanical or biological prosthesis. One patient had had his aortic valve and ascending aorta replaced separately, leaving the aortic root intact. Distal aortic anastomosis was performed utilizing DHCA in all patients. All surgeons reported having had experiences with playing video games. All of them assumed that VR would allow them to study each patient’s anatomy more precisely and have a more realistic representation of the anatomy. Sixty percent of the surgeons expected that utilization of the VR would not result in a reduction in operation time. Seventy percent of the surgeons assumed that VR would promote surgical simulation.

### 3.2. Assessment of CardioVR in the Planning of Aortic Surgery

**Figure 2:**
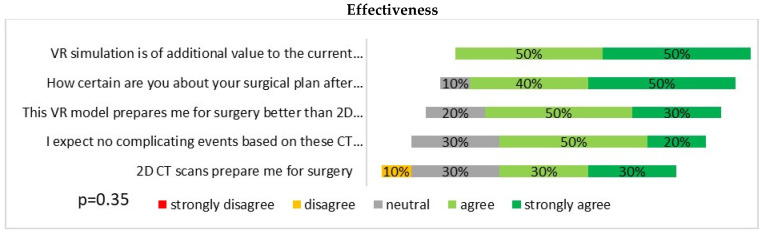
Questionnaire results of participating surgeons on the effectiveness of virtual reality software and hardware for the preoperative planning of ascending aortic surgery.

**Figure 3:**
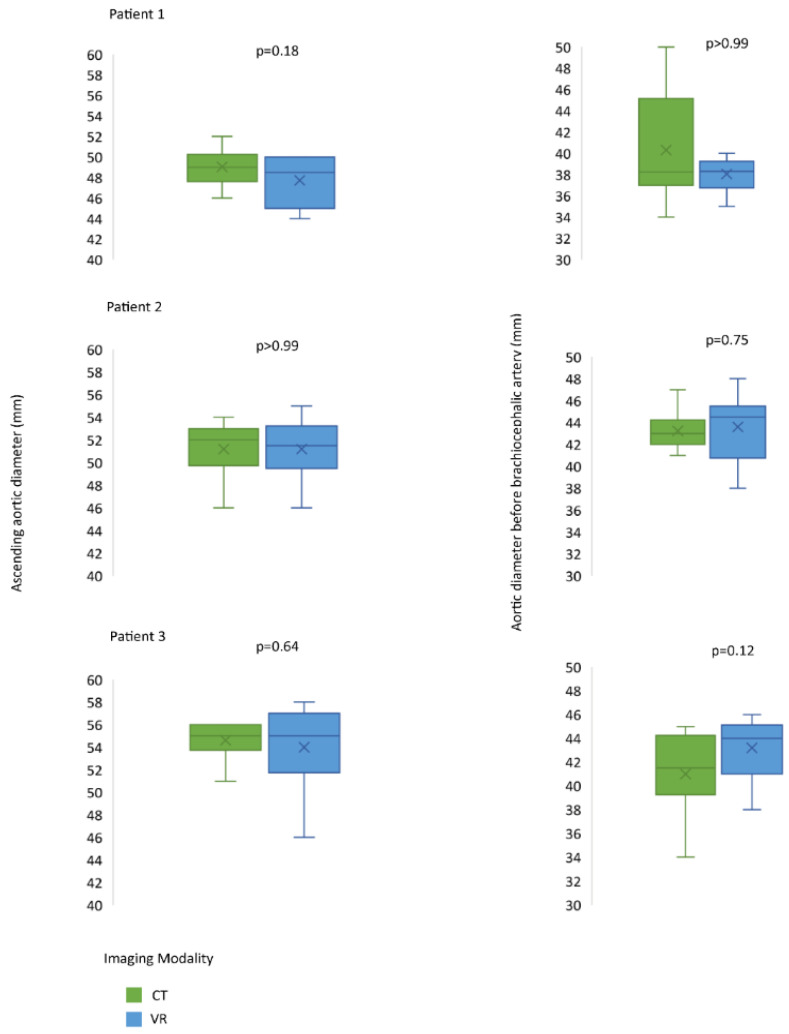

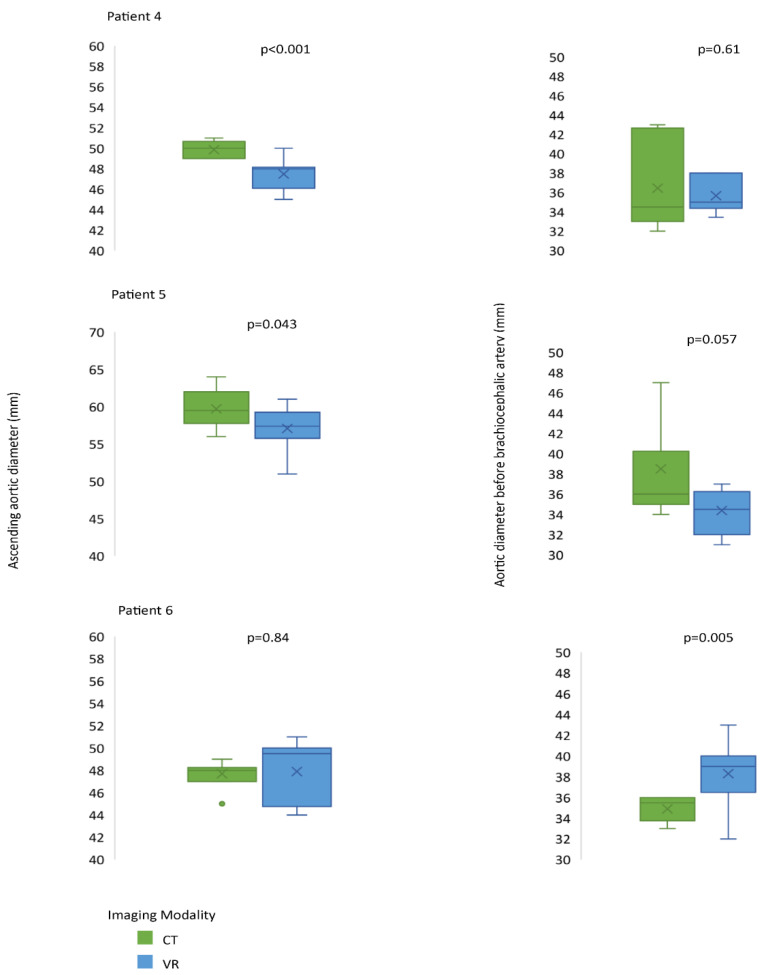
Measurements of maximal ascending aortic diameters and aortic diameters proximal to the brachiocephalic artery.

Based on questionnaire responses, surgeons experienced no significant differences in the effectiveness of preoperative planning with 2D CT scans and VR images (Figure 2). All surgeons agreed that VR simulation played a valuable additional role in the preoperative planning of aortic surgery, and 8 out of 10 agreed that it prepared them better for surgery than 2D CT images. Overall, a good agreement between the measurements in the two modalities was found (Figure 3). VR measurements were on average 1.1 mm lower than CT measurements for the diameter of the ascending aorta, and 0.3 mm lower for the diameter of the aorta proximal to the brachiocephalic artery. In total, after VR guidance, the overall decision about the surgical extent of aortic replacement was altered in two out of six (33%) patients (Table 1).

### 3.3. Evaluation of VR-Guided Aortic Surgery Planning

**Figure 4:**
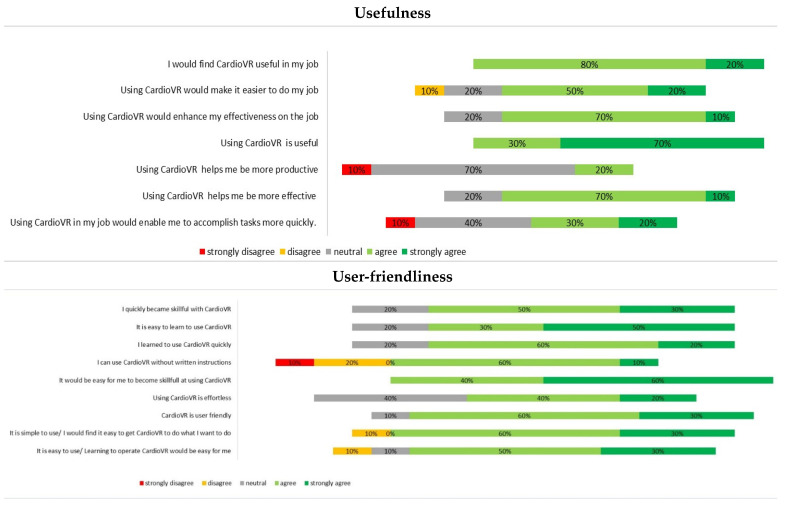

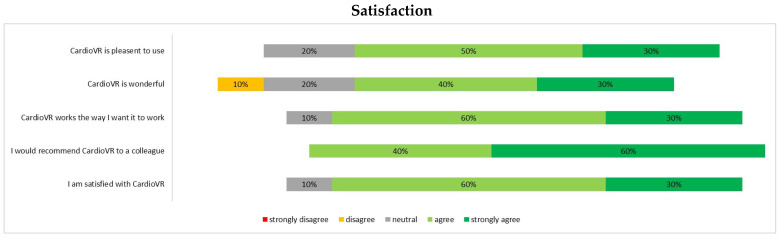
Questionnaire results on the virtual reality experience of participating surgeons for the preoperative planning of ascending aortic surgery.

All surgeons individually evaluated the usefulness and user-friendliness of, and their satisfaction towards, the utilization of VR (Figure 4). The participants rated the aforementioned three topics with median scores of 3.8 (IQR: 3.5–4.1), 4.2 (IQR: 3.8–4.6) and 4.1 (IQR: 3.8–4.7), respectively.

## 4. Discussion

In this study, we found a reported added value of CardioVR in the preoperative assessment of the ascending aorta and aortic arch. Although, based on questionnaire results, we could not find a significant difference in the effectiveness of 2D CT scans and VR images for preoperative planning reported by the surgeons, the majority stated that VR prepared them better for surgery than 2D CT scans.

Attaining a detailed understanding of the surgical anatomy of the ascending aorta and arch can be a challenge based on standard 2D conventional imaging visualizations alone. The challenge lies in the accurate visualization of the course and curvature of the aorta, and its head vessel’s origin. This study indicates that new preoperative 3D VR image processing strategies can help increase surgeons’ understanding of the arch anatomy, thus improving the planning of the extent of resection and optimizing the choice of procedure, such as open distal versus clamped aortic anastomosis. The replacement of the ascending aorta is usually performed with a cross-clamp at the distal ascending aorta. This cross-clamping is one of the routine procedures in cardiac surgery, and the risk from cross-clamping has been lowered to a minimum in regular practice. Anastomosis with a graft adjacent to the cross-clamp is sometimes difficult, especially when the space allowed for the anastomosis is minimal. A remnant of a diseased ascending aorta could dilate over time. Hence, it is natural to consider that a diseased ascending aorta should be replaced completely. In these cases, open distal anastomosis is chosen. Many would argue that, because this leads to hemiarch replacement, it consequently changes the invasiveness of the surgery, carrying additional risk because of the need for DHCA, which has been associated with an increased risk of neurologic dysfunction and hypothermia-induced coagulopathy [11,12,13]. In the end, open distal anastomosis cannot be considered a low-risk procedure for any patient, and the decision to perform a hemiarch replacement must be considered carefully. Therefore, we think that VR immersive imaging may provide additional information during the visualization of the aortic anatomy and, hence, contribute to the application of appropriate procedures and avoidance of unnecessary DHCA when possible.

Furthermore, having a clear preoperative plan leads to effective communication with the surgical team, and enhances the awareness and preparation of the planned procedure for the anesthesiologist, perfusionist, and other operating team members. It also leads to better preoperative information for the patients, providing them with a more accurate plan and promoting patient engagement in surgical planning. Converting existing conventional imaging data into 3D visualizations contributes to overall anatomical understanding [14,15]. Unfortunately, no research on direct comparisons between traditional 2D imaging techniques and VR exists in adult cardiothoracic surgery. Recently, the use of VR in preoperative planning has gained popularity in different surgical fields. A randomized clinical trial performed by Shirk and colleagues [16] showed that the utilization of VR, compared with conventional 2D CT and MRI, during preoperative planning for robotic-assisted nephrectomy in patients with kidney cancer resulted in reduced operative time, estimated blood loss, clamp time, and length of hospital stay. In another study by Lu et al., VR 3D modeling was used to evaluate the application of this technology in the preoperative planning of congenital heart surgery [17]. It showed that, compared with echocardiography and CT/MRI, VR-guided preparation resulted in additional information and also led to an alternation of the surgical plan in two cases (8%). Another value of VR is seen in neurosurgery. Mert and colleagues [18] compared 3D VR with MRI for the preoperative planning of brain tumors. They found that the addition of VR models to standard imaging improved correct tumor lesion localization and intraoperative navigation. At our own department, Sadeghi et al. [19] conducted a prospective observational study on the application of VR for the preoperative planning of video-assisted thoracoscopic segmentectomies. PulmoVR was successfully applied as a supplementary imaging tool and, in 40% of cases, the surgical strategy was adjusted due to the 3D-VR-based evaluation of anatomy.

As the transition from open surgery to minimally-invasive surgery and intervention continues, importance of clear visualization during these procedures and accurate preoperative planning have led to the development of emerging digital techniques. Augmented reality (AR) is another technology that superimposes digital content onto the reality we observe. It allows the wearer to see the native environment while placing 2D or 3D images within it. As AR projects virtual content on top of a real-world environment, it has numerous benefits over VR technology. It can be used during image-guided interventions by overprojecting it over real-time fluoroscopy and can be applied as a navigational guidance tool by the overlapping and fusion of different imaging modalities in open surgical procedures [4,20]. Therefore, this modality might be another promising visualization tool in the near future. Yet another novel technique focuses on computational flow and the parametric modeling of aortic hemodynamics [21,22]. Computational modeling adopts small elements near the aortic wall, resulting in precise wall shear stress predictions when compared with reconstructions derived from cardiac magnetic resonance imaging [23]. Together with novel imaging techniques, these computational flow analyses could further improve our understanding of disease variability and optimize the preoperative planning of ascending aortic surgery.

We have explored intermodal variability in measurements and, although no significant differences were found between the 2D and 3D visualizations in our study, all surgeons agreed that VR played a valuable additional role in preoperative planning. In two cases (33%), the overall decision was changed after seeing the VR images, from performing an open distal anastomosis to clamping the ascending aorta, hence eliminating the need for DHCA. These findings reinforce the statement that 3D VR images can be helpful in providing a comprehensive overview of aortic arch anatomy, facilitating the optimal anatomic localization of the pathology and adjacent structures. The novel 3D immersive VR imaging technique presented here may be a value-added tool when planning complex aortic arch surgery.

### Limitations

For the preoperative planning of endovascular aortic procedures, 3D CT reconstructions have been extensively validated, measuring the inner and outer diameters of the aortic arches and enhancing our understanding of complex anatomy. Hence, 3D CT aortic reconstructions are commonly performed at our department for complex, extensive aortic procedures. Since no 3D CT reconstructions were performed for the patients included in this study, no comparison with 3D CT reconstructions was carried out. Unfortunately, due to our hospital referral system, there was variety in the scanning settings, which in an ideal situation should be avoided. Furthermore, interpretations of these results must be taken with caution due to their observational nature and the limited number of included patients.

VR is a promising tool for future implementation in aortic surgical planning. Nevertheless, challenges still exist for the clinical application of the proposed novel 3D VR technique. To further objectify these data with scientific accuracy, prospective validation is required, with comparisons with actual decisions during surgery followed by prospective (randomized) clinical studies. This study is hypothesis generating only, and further research is needed before we can apply CardioVR in daily practice. In addition, our VR platform does not support MRI. In the future, VR platforms could have a broader impact by enabling the immersive visualization of MRI and ultrasound images, since high-quality and optimal imaging is key for obtaining functional preoperative 3D imaging. In the present study, the segmentation was performed manually with close collaboration between an information technology expert and a cardiothoracic surgeon. The future development of more advanced and (semi-)automatic segmentation could standardize these essential steps with the ultimate goal of automatically visualizing patient-specific anatomy in 3D VR.

Furthermore, intra- and interobserver variability in measurements can serve as an additional source bias and should be explored in the future.

## 5. Conclusions

Our study demonstrates that the immersive 3D VR visualization of aortic anatomy is a potentially beneficial supplementary tool, providing a real-life format for preoperative planning in ascending aortic surgery. This novel form of surgical planning could help improve a surgeon’s understanding of their patient’s anatomy and optimize the surgical approach.

## Figures and Tables

**Table 1:** Overall preference (n = 10) for clamped versus open distal ascending aortic anastomosis per patient based on computed tomography or virtual reality.

	Preference for Open Approach n (%)	Final Preference	Operation Performed Initially
**Patient 1**			
**CT1**	10 (100)	Open	Open
**VR1**	8 (80)	Open	
**Patient 2**			
**CT2**	8 (80)	Open	Open
**VR2**	8 (80)	Open	
**Patient 3**			
**CT3**	10 (100)	Open	Open
**VR3**	9 (90)	Open	
**Patient 4**			
**CT4**	7 (70)	Open	Open
**VR4**	4 (40)	Clamp	
**Patient 5**			
**CT5**	5 (50)	50–50	Open
**VR5**	2 (20)	Clamp	
**Patient 6**			
**CT6**	1 (10)	Clamp	Open
**VR6**	2 (20)	Clamp	

Values are presented as n (%) of the total participating surgeons. CT, computed tomography; VR, virtual reality.

## Data Availability

Datasets used and analysed during the current study are available from the corresponding author on reasonable request.

## Supplementary Materials

The following supporting information can be downloaded at: https://www.mdpi.com/article/10.3390/jcdd9020031/s1, Pre-study Questionnaire. Video S1: Assessment of ascending aorta and aortic arch in virtual reality.
