# Supplementary material for: Virtual Reality in the Preoperative Planning of Adult Aortic Surgery: A Feasibility Study
Source: J Cardiovasc Dev Dis. 2022 Jan 18;9(2):31. doi: 10.3390/jcdd9020031 (PMC8879426; doi:10.3390/jcdd9020031)
Supplement: Supplementary file 1 [file jcdd-09-00031-s001.zip › jcdd-1515022-supplementary/Supplementary material/Questionnaires_Supplementary Material.pdf]

Pre-study Questionnaire

1. Have you played computer games or participated in virtual simulations before?
   Yes/No
2. What do you want Virtual Reality to do for you?

   - Allow to precisely study patients' anatomy Yes/No
   - Save time Yes/No
   - More realistic, precise and accurate representation of anatomy Yes/No
   - Promote surgical simulation Yes/No
   - Provide additional information Yes/No
   - Other……………………………………………………………………………….

3. Have you modified your surgical plan after you started operating on a patient with an ascending aortic aneurysm recently? If yes, what was the proportion of cases in the last year.

Effectiveness Questionnaire

1. This 2D CT scan prepares me for surgery
   1-2-3-4-5 (Strongly disagree → Strongly agree)
2. I expect no complicating events based on this scan
   1-2-3-4-5 (Strongly disagree → Strongly agree)
3. This VR model prepares me for surgery better than 2D CT images
   1-2-3-4-5 (Strongly disagree → Strongly agree)
4. How certain are you about your surgical plan after seeing the VR images?
   1-2-3-4-5 (Very uncertain → Very certain)
5. This VR model is of additional value to the current imaging techniques for decision making in the preoperative planning
   1-2-3-4-5 (Surely not → Surely yes)

USE Questionnaire: Usefulness, Satisfaction, and Ease of use

<u>Usefulness</u>

1. Using CardioVR in my job would enable me to accomplish tasks more quickly.
   1-2-3-4-5 (Strongly disagree → Strongly agree)
2. Using CardioVR helps me be more effective

1-2-3-4-5 (Strongly disagree → Strongly agree)

3. Using CardioVR helps me be more productive

   1-2-3-4-5 (Strongly disagree → Strongly agree)

4. Using CardioVR is useful

   1-2-3-4-5 (Strongly disagree → Strongly agree)

5. Using CardioVR would enhance my effectiveness on the job

   1-2-3-4-5 (Strongly disagree → Strongly agree)

6. Using CardioVR would make it easier to do my job

   1-2-3-4-5 (Strongly disagree → Strongly agree)

7. I would find CardioVR useful in my job

   1-2-3-4-5 (Strongly disagree → Strongly agree)

Ease of use

1. It is easy to use/ Learning to operate CardioVR would be easy for me

   1-2-3-4-5 (Strongly disagree → Strongly agree)

2. It is simple to use/ I would find it easy to get CardioVR to do what I want to do

   1-2-3-4-5 (Strongly disagree → Strongly agree)

3. CardioVR is user friendly

   1-2-3-4-5 (Strongly disagree → Strongly agree)

4. Using CardioVR is effortless

   1-2-3-4-5 (Strongly disagree → Strongly agree)

5. It would be easy for me to become skillful at using CardioVR

   1-2-3-4-5 (Strongly disagree → Strongly agree)

6. I can use CardioVR without written instructions

   1-2-3-4-5 (Strongly disagree → Strongly agree)

Ease of learning

1. I learned to use CardioVR quickly

   1-2-3-4-5 (Strongly disagree → Strongly agree)

2. It is easy to learn to use CardioVR

   1-2-3-4-5 (Strongly disagree → Strongly agree)

3. I quickly became skillful with CardioVR

   1-2-3-4-5 (Strongly disagree → Strongly agree)

Satisfaction

1. I am satisfied with CardioVR
   1-2-3-4-5 (Strongly disagree → Strongly agree)
2. I would recommend CardioVR to a colleague
   1-2-3-4-5 (Strongly disagree → Strongly agree)
3. CardioVR works the way I want it to work
   1-2-3-4-5 (Strongly disagree → Strongly agree)
4. CardioVR is wonderful
   1-2-3-4-5 (Strongly disagree → Strongly agree)
5. CardioVR is pleasant to use
   1-2-3-4-5 (Strongly disagree → Strongly agree)
